# Analysis of Thisbe and Pyramus functional domains reveals evidence for cleavage of *Drosophila *FGFs

**DOI:** 10.1186/1471-213X-10-83

**Published:** 2010-08-05

**Authors:** Sarah Tulin, Angelike Stathopoulos

**Affiliations:** 1 Division of Biology, California Institute of Technology, 1200 E. California Blvd. MC 114-96, Pasadena, California, USA

## Abstract

**Background:**

As important regulators of developmental and adult processes in metazoans, Fibroblast Growth Factor (FGF) proteins are potent signaling molecules whose activities must be tightly regulated. FGFs are known to play diverse roles in many processes, including mesoderm induction, branching morphogenesis, organ formation, wound healing and malignant transformation; yet much more remains to be learned about the mechanisms of regulation used to control FGF activity.

**Results:**

In this work, we conducted an analysis of the functional domains of two *Drosophila *proteins, Thisbe (Ths) and Pyramus (Pyr), which share homology with the FGF8 subfamily of ligands in vertebrates. Ths and Pyr proteins are secreted from *Drosophila *Schneider cells (S2) as smaller N-terminal fragments presumably as a result of intracellular proteolytic cleavage. Cleaved forms of Ths and Pyr can be detected in embryonic extracts as well. The FGF-domain is contained within the secreted ligand portion, and this domain alone is capable of functioning in the embryo when ectopically expressed. Through targeted ectopic expression experiments in which we assay the ability of full-length, truncated, and chimeric proteins to support cell differentiation, we find evidence that (1) the C-terminal domain of Pyr is retained inside the cell and does not seem to be required for receptor activation and (2) the C-terminal domain of Ths is secreted and, while also not required for receptor activation, this domain does plays a role in limiting the activity of Ths when present.

**Conclusions:**

We propose that differential protein processing may account for the previously observed inequalities in signaling capabilities between Ths and Pyr. While the regulatory mechanisms are likely complex, studies such as ours conducted in a tractable model system may be able to provide insights into how ligand processing regulates growth factor activity.

## Background

Fibroblast Growth Factors (FGFs) comprise a large family of signalling molecules that are key regulators of developmental processes including mesoderm induction, gastrulation, cell migration, midbrain-hindbrain patterning, limb induction and bone formation [1-7]. FGFs continue to function in adult tissue homeostasis and wound healing; when improperly activated they can also contribute to many human diseases and cancer [7-10]. Most of the 24 known FGF ligands in vertebrates are small proteins with a molecular mass of 17-34 kD, whereas the three known *Drosophila *FGF ligands are all predicted to be much larger proteins with molecular masses of approximately 80 kD [11,12]. Vertebrate FGFs and *Drosophila *FGFs share homology within their FGF domains, but *Drosophila *FGFs have an additional long, low-complexity sequence of unknown function.

The FGF ligands in *Drosophila *are Branchless (Bnl), Thisbe (Ths), and Pyramus (Pyr), and they bind to FGF receptors (FGFR), which are receptor tyrosine kinases (RTKs). FGF signalling is used pervasively throughout development. Bnl-mediated activation of the Breathless (Btl) receptor controls branching of the developing trachea [13], while Ths and Pyr activate the Heartless (Htl) receptor to control movement of the mesoderm cells[14-18], pericardial cell specification[15,16,18,19], and caudal visceral mesoderm migration [20,21]. Pyr and Ths ligands also function later in development within the nervous system to control glial cell proliferation, migration and axonal wrapping [22]. Ths and Pyr are thought to share one receptor, which makes *Drosophila *an ideal model to study FGF signaling specificity and differential regulation. Initial work on the individual functions of Ths and Pyr in the embryo was recently described using genetic approaches, where it was found that although both ligands play a role in mesoderm spreading, Pyr is more important for pericardial cell specification [18,19].

In order to achieve a better understanding of how Ths and Pyr proteins are adapted to their particular roles, it is necessary to first understand the mechanism by which signaling with a particular FGF ligand occurs, and the way this signaling is regulated. Signaling ligands can be intracellular, membrane-bound, or secreted, and are often modified and processed in many different ways. Understanding these basic properties of a signaling ligand provides important clues for any further mechanistic studies.

Proteolytic processing is a common regulatory mechanism of growth factors and other signaling pathways in both vertebrates and *Drosophila*. Examples from *Drosophila *include the EGF ligand Spitz (Spi), TGF-β ligands Decapentaplegic (Dpp) and Glass Bottom Boat (Gbb), Spätzle, Notch, and Delta. Spi is cleaved in its transmembrane domain to release a secreted form (sSpi) that can bind to the *Drosophila *EGF Receptor (DER) [23,24]. The Spätzle C-terminal cysteine knot is activated when cleaved away from an unstructured, inhibitory N-terminal domain [25-27]. Dpp and Gbb, like their vertebrate BMP homologs, are produced as inactive preproproteins and cleaved by Furin1 and Furin2 to release the mature, active protein [28]. Notch is produced as a single polypeptide but is then processed in the secretory pathway by a furin-like protease within the Golgi to produce two fragments that remain non-covalently associated [29-31]. Lastly, Delta undergoes three proteolytic cleavages and one of these cleavages is dependent on the ADAM metalloprotease Kuzbanian [32]. Uncovering the proteolytic processing events of these growth factors and signaling molecules has led to a deeper understanding of their signaling mechanism and regulation.

Here we have found evidence for (1) the proteolytic cleavage of Ths and Pyr full-length precursor proteins and (2) the secretion of the FGF-domain-containing N-terminus. The role of proteolytic processing in FGF signaling is currently limited to one vertebrate FGF ligand, FGF23, which is part of a subgroup of endocrine FGFs. Full length FGF23 is 251 amino acids and is cleaved by subtilisin-like proprotein convertases between amino acids 179 and 180. In humans, failure of this cleavage step results in secretion of additional full-length FGF23, which can cause hypophosphatemia leading to autosomal dominant hypophosphatemic rickets/osteomalacia [33,34]. These studies support the view that a delicate balance is necessary to control the level of secreted bioactive FGF proteins [35]. We also show that after processing, Ths and Pyr are similar in size to their vertebrate homolog FGF8 (~G30 kD) suggesting that studying regulation of FGF signaling in *Drosophila *could provide useful insights for the FGF field in general.

In addition to understanding the processing of Ths and Pyr, we sought to link the structural domains to the function of the ligands. From embryonic stage 10 to 11, the developing dorsal mesoderm requires activation of the Htl receptor to specify two *even-skipped *(*eve*) expressing progenitor cells, which give rise to three Eve-positive founder cells [36]. Two of these Eve-positive founders will become Eve-positive pericardial cells, and the third founder will give rise to dorsal somatic muscle [37-39]. When either Ths or Pyr are ectopically expressed throughout the neurogenic ectoderm using a 69B-GAL4 driver, the Eve-positive cell cluster increases from three cells to as many as 20 cells [16,18]. In this work we used these supernumerary Eve-positive cells as a functional readout of Ths and Pyr activity. By analyzing a series of truncation, deletion, and chimeric constructs, our results collectively suggest that the N-terminal FGF domain alone is sufficient to support function, but only when properly folded and secreted.

If the N-terminus alone is able to activate the receptor and allow downstream signaling, then what is the role of the long C-termini of Ths and Pyr? We addressed this question with another GAL4 driver, ZenKr-GAL4, which drives expression only in a subset of the dorsal ectoderm of the early embryo (i.e., zenVRE.Kr-GAL4 [40]). Limiting the source of protein production to this restricted domain allowed us to assay differences in the range-of-action of different Ths and Pyr constructs. Our results suggest that the Ths C-terminus is inhibitory and the Pyr C-terminus is not. Collectively, these findings demonstrate that post translational processing is important for FGF signaling during embryonic development of *Drosophila *and suggest that processing of signaling ligands may be widespread.

## Results

### Comparison of predicted protein characteristics for Thisbe and Pyramus

A screen to uncover genes involved in patterning the dorsal-ventral axis of *Drosophila*, identified expression of the *thisbe *gene (*ths*: previously called *Neu4*) in the neurogenic ectoderm [41]. Results from additional genetic experiments were consistent with the hypothesis that Ths and Pyr are two FGF ligands for the Htl FGF receptor [15,16]. To understand the mechanism of FGF signaling through Htl on a molecular level, we characterized Ths and Pyr proteins by analysing their functional domains. We first considered predictions about the size and homologous domains of Ths and Pyr.

**Figure 1:**
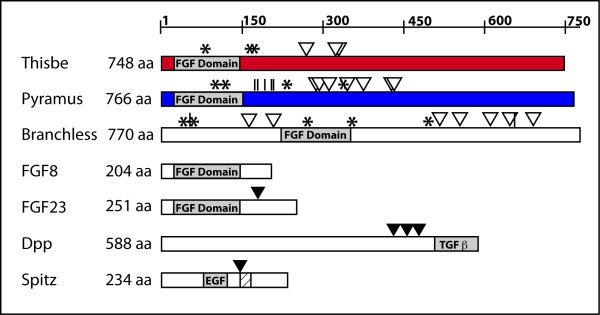
**Comparison of Ths and Pyr Proteins to other signalling ligands**. *thisbe *and *pyramus *genes encode proteins of 748 and 766 amino acids (aa), respectively, making them far larger than their vertebrate homolog FGF8, which is 204 aa. Branchless, another FGF ligand in *Drosophila*, is also a relatively large protein of 770 aa. The C-terminus is cleaved from FGF23, the only FGF family member known to be cleaved [33,34]. Dpp is produced as a 588 aa precursor, but is cleaved to primarily the TFG-β-homologous domain alone [23,28,45,46]. Spitz is processed within its transmembrane domain and, like Thisbe and Pyramus, binds to a RTK receptor to signal [23,24]. Known cleavage sites are marked with a black inverse triangle. In the *Drosophila *FGFs, potential cleavage sites consisting of multi-basic amino-acid motifs are marked with a white inverse triangle. Predicted N-glycosylation sites are marked with an asterisk and predicted O-glycosylation sites are marked with a vertical line.

The *ths *cDNA contains a 2,247 basepair open-reading frame and is predicted to encode a protein of 748 amino acids (aa) with a molecular weight of 82.2 kD. Thisbe is predicted to have a N-terminal signal peptide followed by a 122 aa FGF domain composed of 12 predicted β-strands separated by coiled-coil domains, which presumably support a trefoil structure like vertebrate FGFs. Beyond the FGF domain, however, the C-terminal domain of Ths exhibits only limited homology within deuterostomes, to other uncharacterized "immunoglobulin-like proteins" or proteins that are known to be highly glycosylated (data not shown). The Ths protein sequence also contains several dibasic and multi-basic motifs characteristic of the recognition site for furin proteases [42,43] and several predicted N-linked glycosylation sites (Fig. 1).

The *pyr *cDNA contains a 2,301 basepair open-reading frame and is predicted to encode a protein of 766 aa and ~87 kD. Pyr also has a N-terminal signal peptide followed by a FGF domain of 128 aa (Fig. 1). Ths and Pyr share 39% amino acid identity in the FGF core domain. C-terminal to the Pyr FGF domain, there are many repeats and regions of low complexity. From amino acids 399 to 426, Pyr has a string of hydrophobic amino acids that weakly qualifies as a potential transmembrane domain when assayed by topology prediction programs using the Kyte-Doolittle Scale [44]. Pyr also has sites of predicted N-linked and O-linked glycosylation and putative dibasic and multi-basic protease recognition sites (see symbols in Fig. 1).

### Ths and Pyr are secreted from S2 cells and detectable as cleaved forms

**Figure 2:**
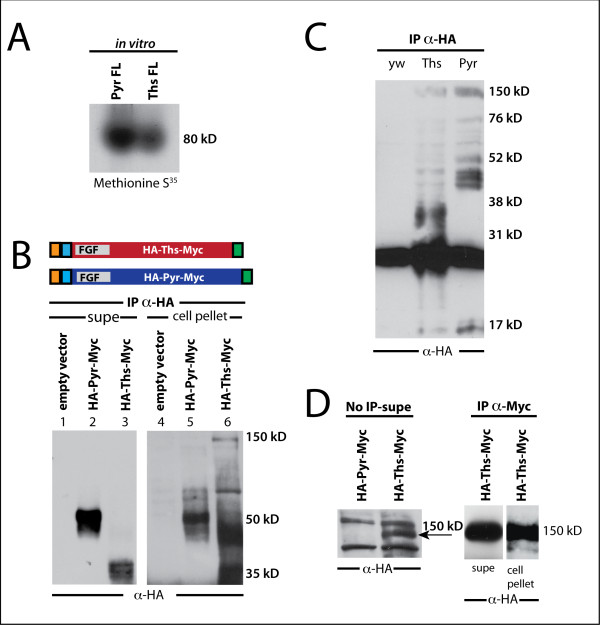
**FGFs are cleaved in S2 cell culture and embryonic extracts**. (A) *In vitro *transcription/translation incorporating S^35^Met into Pyr (left) and Ths (right) supports production of ~80 kD proteins, as predicted from the sequence. (B) Schematics of HA-Pyr-Myc and HA-Ths-Myc constructs showing the position of the signal sequence (orange box), N-terminal HA-tag (blue box), FGF domain, and C-terminal Myc tag (green box). Upon transfection of S2 cells, HA-Pyr-Myc and HA-Ths-Myc are secreted from cells as multiple bands around 50 kD for Pyr (lane 2) and 35 kD for Ths (lane 3), as detected by immunoprecipitation and immunoblot with anti-HA to track N-termini. Lane 1 and 4 are supernatant and cell pellet controls transfected with empty vector (i.e., pUASt). Lane 5 and 6 are immunoprecipitations of HA-Pyr-Myc and HA-Ths-Myc from the cell pellet, showing cleaved forms are already detectable inside the cell. (C) Extracts from wildtype embryos (yw) or embryos overexpressing HA-Ths (Ths) or HA-Pyr (Pyr) with the 69B-GAL4 driver, immunoprecipitated with rat anti-HA and detected with mouse anti-HA reveal cleaved bands around 35 kD for Ths and around 45 kD for Pyr. (D) (Left Blot) Supernatant (i.e., cell culture medium) from HA-Pyr-Myc and HA-Ths-Myc, without immunoprecipitation, blotted with anti-HA antibody, shows a full-length band in the supernatant for Ths but not Pyr; the full-length Ths protein is present at much lower levels than the cleaved form and is only observable upon longer exposure; for instance, in (B), lane 3, it is not detected. (Right Blot) Immunoprecipitating with anti-Myc and blotting with anti-HA shows that the 150 kD band in Ths supernatant and cell pellet has both the N- and C-terminus connected.

To confirm the ability of the full-length *ths *and *pyr *cDNAs to support the production of ~80 kD proteins as predicted by their sequence, we expressed Ths and Pyr proteins *in vitro *using a rabbit reticulocyte transcription/translation kit that incorporates S^35^-labeled Methionine. Full-length proteins were detected at ~80 kD, as predicted (Fig. 2A).

We compared the size of Ths and Pyr proteins with other ligands (e.g., Bnl, FGF8, Dpp, Spi, and FGF23; see Fig. 1), and found Ths and Pyr to be much larger than FGF8 and closer in size to other cleaved growth factors in *Drosophila *like Dpp. Therefore, we hypothesized that Ths and Pyr may also be regulated by cleavage. Dpp (588aa) is activated by cleavage into much smaller molecules consisting primarily of the TGFβ-homologous domain [28,45,46]. Spitz is an EGF ligand that, like Ths and Pyr, uses a high-affinity RTK receptor to signal. Spitz is cleaved within its transmembrane domain to release the EGF domain as a small secreted ligand [23,24]. All FGFs in vertebrates, even the cleaved FGF23, are small molecules consisting mostly of the FGF domain alone. These comparisons led us to consider the hypothesis that Ths and Pyr may not signal as long, full-length proteins, but as small molecules consisting primarily of the FGF domain.

First, we sought to verify whether Ths and Pyr were indeed secreted proteins by transiently expressing *ths *and *pyr *from a metallothionine promoter in S2 cells, a cell line derived from *Drosophila *embryonic cells. Double mutants for *ths *and *pyr *genes (i.e. Def(2R)BSC25 [16]) phenocopy the *htl *mutant phenotype, but in the early *Drosophila *embryo Ths and Pyr proteins are expressed in the ectoderm while Htl is limited to the abutting mesoderm cells. Thus, for Ths and Pyr to influence the activity of the Htl FGFR in the mesoderm, our working hypothesis had been that the FGF ligands are secreted from the ectoderm to activate the FGFR present in the mesoderm. Consistent with this view, signal sequences are predicted at the N-terminus within the identified protein sequences [16]. Nevertheless, we sought to examine secretion directly. In order to follow both the N- and C-termini separately, we constructed epitope-tagged constructs with a single hemagglutinin (HA) tag at the N-terminus after the signal peptide and a 6X Myc tag at the C-terminus (diagrams in Fig. 2B). UASt.HA-Ths-Myc and UASt.HA-Pyr-Myc plasmids were co-transfected into S2 cells along with the metallothionine-inducible Gal4 plasmid and ectopic expression of the tagged proteins was achieved by copper induction. Using anti-HA antibody, we were able to immunoprecipitate N-terminally tagged Ths and Pyr from the culture medium, demonstrating directly for the first time that the proteins are indeed secreted (Fig. 2B, lanes 2 and 3).

Instead of identifying secreted proteins at the predicted full-length molecular weights, we found the predominant secreted forms consisted of multiple bands running at ~35 kD for Ths and ~50 kD for Pyr (Fig. 2B, lane 2 and 3, respectively), indicating that the cleaved N-terminus of each protein is secreted.

### Cleaved forms of Ths and Pyr are detected in embryonic extracts

To investigate whether Ths and Pyr proteins are also cleaved in the animal, we expressed tagged versions of these proteins (i.e., pUASt-HA-Ths and pUASt-HA-Pyr) in the embryo using the pan-ectodermal driver 69B-GAL4. Embryonic extracts were prepared from 1 gram of collected embryos, age 0-24 hour, and N-terminal protein species were isolated by using an anti-HA antibody for immunoprecipitation. Cleaved forms of both Ths and Pyr were detected in these samples, at ~35 kD for Ths and ~45 kD for Pyr (Fig. 2C). The ability to detect cleavage products from embryonic extracts of approximately the same size as those secreted in S2 cells suggests our analysis of Ths and Pyr processing in S2 cells may also be relevant to FGF function in the *Drosophila *embryo.

### The C-terminal domain of Ths can be secreted, but not that of Pyr

Our ability to detect cleaved products in the S2 cell culture system, similar in size to those present in the animal, gave us confidence that we could use cell culture to obtain additional insights into these proteins. Therefore, we also examined the cell pellet fractions and found that cleaved N-terminal domains of Ths and Pyr are present inside the cell as well (Fig. 2B, lane 5 and 6). This result suggests that cleavage occurs inside the cell. To examine this possibility more closely, we assayed for the presence of full-length forms of Ths or Pyr inside and outside the cells.

In the HA-Ths-Myc cell pellet sample, in addition to the smaller potentially cleaved forms of Ths, we also detected a polypeptide of 150 kD, one that is much larger than the predicted size for Ths protein (~80 kD) or that is observed when the cDNA is translated *in vitro *(Fig. 2B, lane 6, compare with Fig. 2A). To confirm that this protein species represented the full-length form of Ths, we immunoprecipitated with anti-Myc and blotted with anti-HA to identify both the C- and N-termini simultaneously. We observed that both N- and C-termini were connected in the 150 kD band in both the supernatant and cell pellet (Fig. 2D). Therefore, the 150 kD band probably represents full-length Ths, likely modified by glycosylation or other modifications that retard its mobility when assayed by SDS-PAGE and Western blot. Collectively, these results are consistent with the idea that the majority of Ths is cleaved intracellularly and secreted, while some full-length form is also secreted at lower levels.

### Subceullular Localization of Pyr and Ths

**Figure 3:**
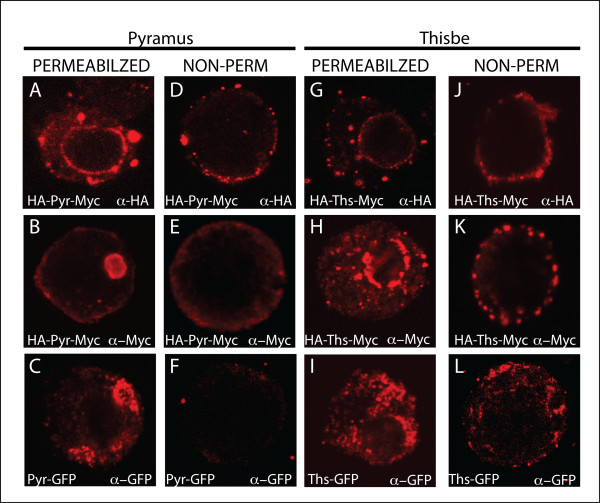
**Visualization of N- and C-termini in S2 cells shows a difference in the subcellular localization of Ths and Pyr C-terminus**. S2 cells were transfected with the indicated pUASt.HA-Pyr-Myc, pUASt.Pyr-GFP, pUASt-Ths-GFP or pUASt.HA-Ths-Myc constructs (see methods), and immunofluorescence was conducted using anti-HA, anti-Myc, or anti-GFP antibodies (all "red"). HA-Pyr-Myc was stained with anti-HA to see the N-terminus (A,D) where predominant ER staining was seen inside the cell (A) while only membrane staining was seen under non-permeabilizing conditions (D). The C-terminus of Pyr was visualized with anti-Myc for HA-Pyr-Myc (B,E) or anti-GFP for Pyr-GFP (C,F). The C-terminus of Pyr inside the cell was localized to small, non-nuclear vesicles, which may be endosomal in character (B,C). No Pyr C-terminus was visualized outside of the cell (E,F). Anti-HA was used to visualize the HA-Ths-Myc N-terminus under permeabilizing (G) and non-permeabilizing conditions (J), revealing ER staining around the nucleus inside the cell (G) and proteins attached to the cell membrane (J). Anti-Myc (H,K) and anti-GFP (I,L) were both used to visualize the Ths C-terminus of either HA-Ths-Myc or Ths-GFP. Again, ER staining was seen inside the cell (H,I) and membrane staining was observed under non-permeabilized conditions (K,L).

We were not able to immunoprecipitate the predicted full-length Pyramus protein from the cell pellet or supernatant using a combination of anti-HA and anti-Myc antibodies, nor could we detect the cleaved C-terminus of Pyr by Western blot using an anti-Myc antibody; in contrast, full-length Ths could be detected in the supernatant at 150 kD even in the absence of immunoprecipitation (Fig. 2D). Possible interpretations of these results are that (1) Pyr protein is processed from full-length very quickly intracellularly, (2) that in S2 cells Pyr protein is never made as a "full-length" form, or alternatively, (3) the Myc epitope is not accessible. To address this question, we stained S2 cells expressing HA-Pyr-Myc constructs with either anti-HA or anti-Myc antibodies (Fig. 3). The stainings provided support that the N-terminus of Pyr (marked by the HA-tag) is secreted from the cell, as staining at the cell periphery was observed even in the absence of cell permeabilization (Fig. 3A,D). In contrast, the anti-Myc staining suggested that the C-terminus of Pyramus is present solely within cells, within an unidentified organelle, possibly an endosome (Fig. 3B). No anti-Myc staining could be observed for HA-Pyr-Myc in the absence of permeabilization (Fig. 3E). As a control for the accessibility of the Myc epitope, we used a C-terminally fused Pyr-GFP construct and anti-GFP antibody to confirm the location of the Pyr C-terminus. The anti-Myc and anti-GFP stainings of HA-Pyr-Myc and Pyr-GFP, respectively, exhibit the same intracellular staining that is lost in the absence of permeabilization (Fig. 3B,C,E,F). This data suggests that the C-terminus of Pyramus is translated, but that the full-length and C-terminus of the protein stays within the cell and is not secreted. Stainings for Ths confirmed what was seen in Western blots. The N-terminus was present both inside the cell (Fig. 3G) and at the membrane in non-permeabilized cells (Fig. 3J). Stainings marking the C-terminus of Thisbe with anti-Myc and anti-GFP support the idea that the C-terminus Thisbe protein is secreted, although this could represent either a full-length or cleaved form (Fig. 3H,I,K,L). Therefore, we propose that there may be a difference in the number of forms secreted for Ths versus Pyr: Ths may be secreted as both a full-length and a cleaved form, whereas Pyr is only secreted as a cleaved form with the C-terminus being retained intracellularly.

### Truncation constructs reveal the FGF domain alone is sufficient for function

In order to reconcile the biochemical evidence for cleaved forms with the endogenous function in the embryo, we made a series of N-terminally, HA-tagged truncation constructs and used the site-directed transgenic method to insert all transgenes in the same genomic location on the third chromosome, 86FB, to minimize positional effects [47]. We cannot be sure that the act of truncation itself does not impart differences in the stabilities of the produced proteins; in fact, stability of the proteins (possibly regulated by cleavage events) may be one mechanism by which their activities are regulated. However, by minimizing positional effects on the transgene, we standardized expression levels for each of the constructs to the best of our abilities.

**Figure 4:**
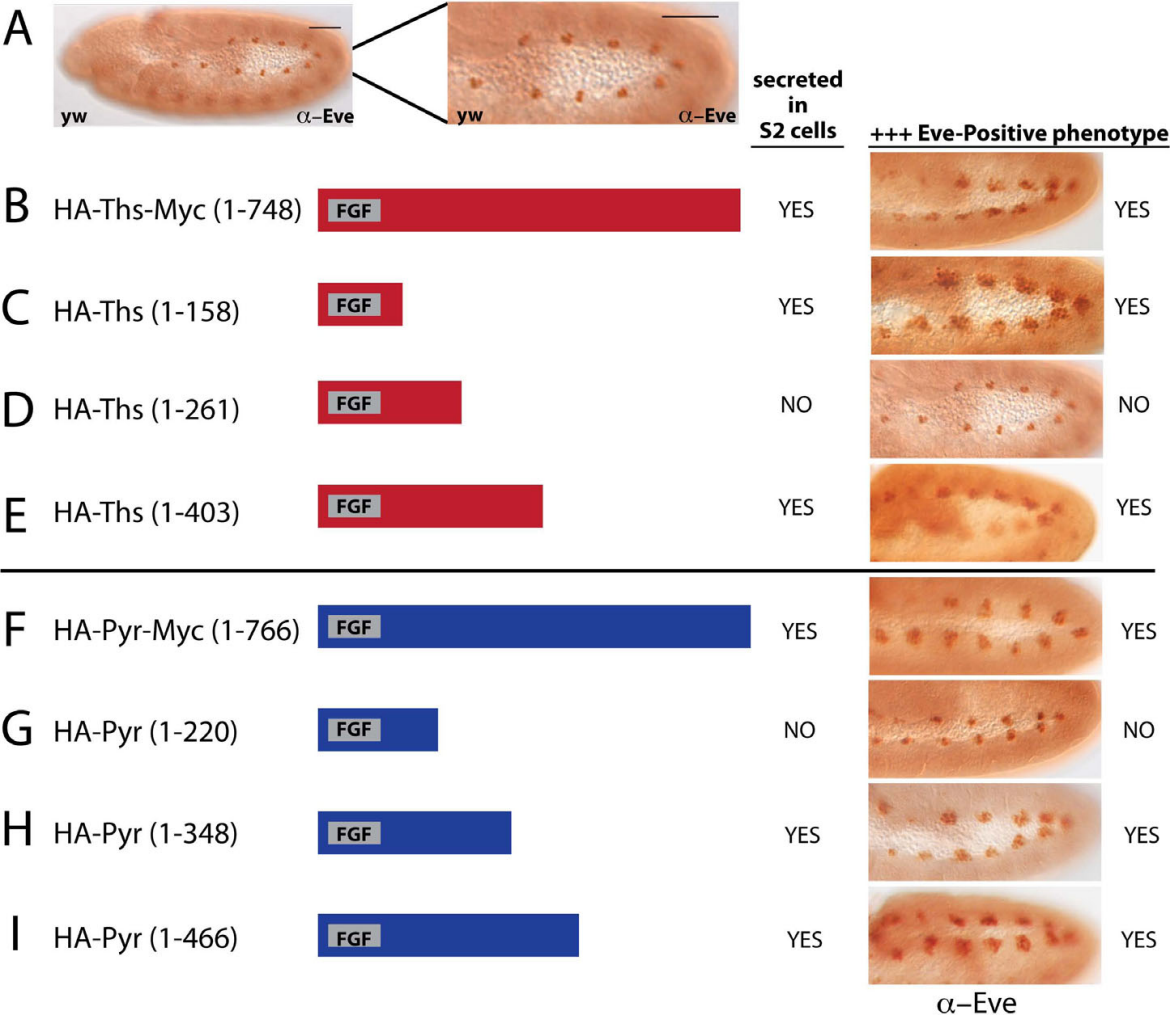
**Ths and Pyr truncation constructs that support the production of secreted proteins in cell culture are also functional in the embryo**. (A) Stage 11, wild type embryos, lateral view, stained with anti-Eve antibody show Eve-positive staining in three cells per hemisegment. The enlargement of the Eve-positive area is 1.8x. (B-I) pUASt-HA-Ths and pUASt-HA-Pyr full-length and truncated construct schematics; assay for secretion was conducted in S2 cell culture. Embryos overexpressing indicated constructs with 69B-GAL4 were stained with anti-Eve antibody to score for FGF activity. (B) Overexpression of full-length Ths results in more Eve-positive cells (Eve+++). (C, E) HA-Ths^1-158 ^and HA-Ths^1-403 ^are both secreted and Eve +++, but (D) HA-Ths^1-261 ^is not secreted and does not support more Eve-positive cells. (F) Overexpression of full length Pyr also results in Eve+++. (G) HA-Pyr^1-220 ^is not secreted and does not give more Eve-positive cells, but (H, I) HA-Pyr^1-348 ^and HA-Pyr^1-466 ^are both secreted and exhibit the Eve+++ phenotype.

During stage 10 to 11, FGF signaling through Htl contributes to differentiation of the mesoderm into specific cell types, including the pericardial cells of the future heart tube and dorsal somatic muscle [36]. At this stage, *pyr*, and to a lesser extent *ths *as well, is expressed in the ectoderm overlying the developing heart cells [16]. Signaling through Htl, presumably by wild type endogenous levels of Pyr/Ths, supports the generation of three Eve-positive cells per hemisegment (Fig. 4A and [48]), while overexpression of either Ths or Pyr leads to the expansion of this cluster up to 20 cells [18].

We used the expansion of the Eve-positive cell cluster as a functional readout to test the function of Ths or Pyr tagged, truncated proteins when overexpressed in the ectoderm with 69B-GAL4. First, addition of HA and Myc tags to Ths and Pyr did not affect the ability of Ths and Pyr to cause an expansion of the Eve-positive cluster (Fig. 4B,F). Furthermore, of three truncation constructs engineered for Ths, two were functional (HA-Ths^1-158 ^and HA-Ths^1-403^) and one was not (HA-Ths^1-261^). The two that were functional were also secreted, as confirmed by expression in S2 cells, while the construct that was not functional was not secreted (Fig. 4C,D,E); the non-functional truncation may disrupt an essential secondary structure required for proper folding and in turn secretion. Remarkably, the small HA-Ths^1-158 ^was secreted and functional, yet this polypeptide contains little more than the FGF domain alone. Together, these data suggest that the FGF domain alone is sufficient for function of Ths and that secretion is also required for function.

Three truncated constructs were engineered for Pyr as well: Pyr^1-220^, Pyr^1-348^, and Pyr^1-466^. Similar to the results from Ths, the two Pyr truncations that were secreted were also functional (Pyr^1-348 ^and Pyr^1-466^) (Fig. 4H,I), while Pyr^1-220 ^was neither secreted nor functional (Fig. 4G). Unlike Ths, the fact that Pyr^1-220 ^was not functional suggests that the shortest functional Pyr construct requires additional sequence besides the FGF domain. It may be possible to make a shorter functional construct of Pyr; the Pyr^1-220 ^construct may have been terminated in a location critical for proper folding. Nevertheless, the functionality of Pyr^1-348 ^suggests aa residues 349-766 are not required for activity.

### Differential range-of-action resulting from limiting the source of FGF

**Figure 5:**
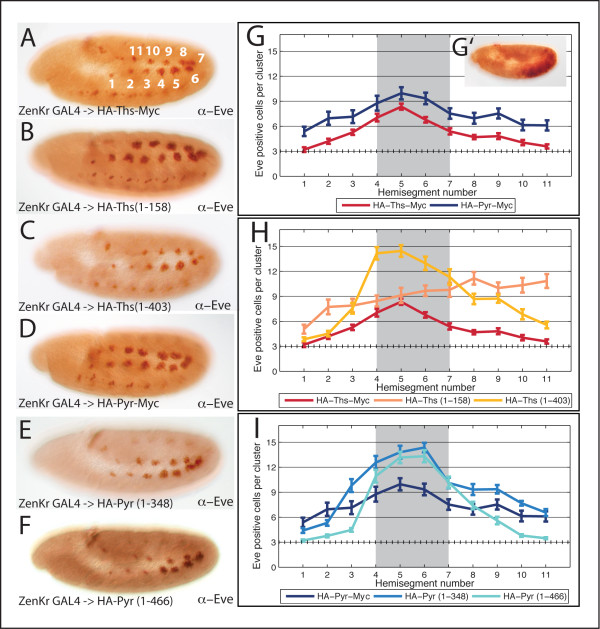
**Restricting the source of full-length and truncated Ths and Pyr constructs reveals a functional difference**. (A-F) Immunohistochemistry on stage 11 embryos, lateral view, all constructs driven with ZenKr-GAL4; embryos were stained using an anti-Eve antibody. (A) White numbers indicate position of numbered Eve-positive clusters; ZenKr-GAL4 supports expression in clusters 4-7. (A-F) Eve staining reveals additional Eve-positive cells outside the ZenKr domain for (A) HA-Ths-Myc (B) HA-Ths^1-158 ^(C) HA-Ths^1-403 ^(D) HA-Pyr-Myc (E) HA-Pyr^1-348 ^(F) HA-Pyr^1-466^. (G,H,I) Eve-positive cells per cluster were counted in each hemisegment for 25 embryos per construct tested and averaged. Error bars indicate standard error. (G) The hatched line at "3" represents the wild-type level of Eve-positive cells. The gray box represents the source of expression supported by ZenKr-GAL4. Plot of Eve-positive cells generated by ZenKr-GAL4 → pUASt-HA-Ths-Myc as compared to ZenKr-GAL4 → pUASt-HA-Pyr-Myc shows that Pyr has greater functional activity than Ths. Ths and Pyr both give a graded output of Eve-positive cells with the most cells in the source domain. (G') ZenKr-GAL4 driving UAS-lacZ and stained with anti-βgal shows the domain of the driver in the posterior dorsal ectoderm of the embryo. (H) ZenKr-GAL4 → pUASt-HA-Ths^1-158 ^does not have the same Eve-positive profile, instead it results in more Eve-positive cells in clusters 8-11. ZenKr-GAL4 → pUASt-HA-Ths^1-403 ^has increased activity locally but similar levels of function to HA-Ths-Myc at long-range (I) ZenKr-GAL4 → pUASt-HA-Pyr^1-348 ^and ZenKr-GAL4 → pUASt-HA-Pyr^1-466 ^both retain a graded profile of Eve-positive cells, although HA-Pyr^1-348 ^supports more Eve-positive cells in distant clusters 8-11 as compared to HA.-Pyr^1-466^.

Previous studies on the function of Ths and Pyr have speculated that a possible difference in their signaling capacity is due to either a differential range-of-action of the ligands diffusing from their source of expression or due to an unequal potency of activating the receptor (e.g., receptor-binding affinity) [18]. In order to address these unanswered questions and to gain more sensitivity than was possible with the pan-ectodermal 69B-GAL4 Eve-positive cluster assay (Fig. 4), we used a different driver, ZenKr-GAL4 which drives expression in a subset of the embryonic dorsal ectoderm starting just before stage 9; at this stage embryos have undergone 50% of germ band elongation and expression supported by the driver is localized to the posterior (Fig. 5G' and zen.VRE.Kr-GAL4 [40]). For each construct, the number of Eve-positive cells per cluster in each hemisegment of 25 embryos was counted, averaged and compared. The clusters were tracked within embryo hemisegments as indicated by the numbers on Fig. 5A. ZenKr-Gal4 supports expression in clusters 4-7 (as seen in inset Fig. 5G') and this domain is represented on the graphs by a shaded gray box in the background.

ZenKr-GAL4 driving full length HA-Ths-Myc and HA-Pyr-Myc both resulted in extra Eve-positive cells outside the expression domain supported by the ZenKr enhancer (i.e., clusters 1-3 and 8-11) (Fig. 5A,D). Furthermore, expression of HA-Pyr-Myc resulted in more Eve-positive cells within every hemisegment as compared with expression of HA-Ths-Myc (Fig. 5G). One interpretation of this result is that Pyr may be more potent in its activation of the Htl receptor and another is that the Pyr protein is secreted at higher levels or is more stable than Ths. Both HA-Pyr-Myc and HA-Ths-Myc supported more Eve-positive cells at the source (i.e., clusters 4-7; gray box) and tapered off in a graded manner to more distant clusters (Fig. 5G).

Expressing HA-Ths^1-158 ^in the ZenKr domain resulted in a surprising result: when compared to full length HA-Ths-Myc, HA-Ths^1-158 ^supported the expression of many more Eve-positive cells in each hemisegment, even those farthest from the source (Fig. 5B,H, especially clusters 1 and 11). Compared to full-length HA-Ths-Myc, HA-Ths^1-158 ^also had a dramatically different profile of Eve-positive cell numbers; instead of peaking at the source and dropping off in a graded manner, there was close to maximum expression of Eve supported in almost every hemisegment (Fig. 5H). In contrast, the other truncated Ths construct, HA-Ths^1-403 ^showed an increase of Eve positive cells as compared to HA-Ths-Myc within clusters at the source yet tapered off in activity in more distant clusters, a profile similar to that of the full length construct (Fig. 5C,H). In summary, two changes in trend were associated with constructs HA-Ths^1-158 ^and HA-Ths^1-403 ^compared with full-length Ths: (I) flattened profile versus (II) increase peak yet graded profile, respectively.

With the overexpression of the ligands limited to the domain of ZenKr-Gal4 expression, we favor the idea that supernumerary Eve-positive cells in hemisegments outside this domain would most likely result from an increase in diffusion of the ligands from their source of ectopic expression or decreased receptor-mediated endocytosis; however we cannot dismiss an alternate scenario in which this result is supported by the Ths^1-158 ^protein being more stable than other constructs. In either case, our results suggest the C-terminus of Ths has an inhibitory function (for example, either affecting stability, endocytosis or diffusion) and possibly that cleavage of Ths plays a regulatory role in increasing the ability of this protein to support activation of FGFR.

We also expressed both functional truncations of Pyr with ZenKr-GAL4 and found that overexpression of both HA-Pyr^1-348 ^and HA-Pyr^1-466 ^supported additional Eve-positive cells (Fig. 5E,F). For both HA-Pyr^1-348 ^and HA-Pyr^1-466 ^the profile was similar to trend II seen for HA-Ths^1-403 ^: increased peak levels but graded profile (Fig. 5I). Our hypothesis is that only the N-terminal Pyr cleavage product is secreted and the C-terminus remains intracellular so truncated Pyr would be expected to support a similar response to expression of full-length Pyr due to essentially the same net protein fragment being secreted. We do indeed see a similar profile of expression (i.e., graded profiles). However, the outputs observed for the two Pyr truncation constructs and full-length Pyr are not identical; we suggest these differences may be due to differences in protein production (e.g., stability, differential processing inside the cell, and/or rate of secretion).

### Pyr truncation constructs are modified

**Figure 6:**
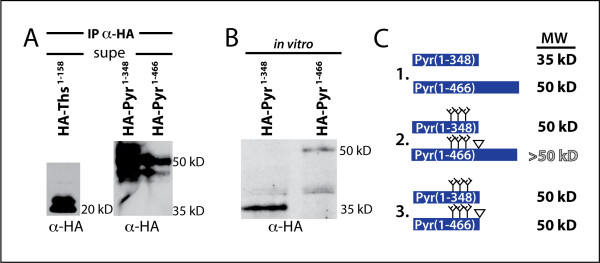
**Post-translational modifications contribute to the observed molecular weight of Pyramus**. (A) Supernatants from S2 cultured cells, immunoprecipitated with anti-HA, blotted with anti-HA. HA-Ths^1-158 ^runs at the expected size of 20 kD, but HA-Pyr^1-348 ^runs larger than predicted, at the same size as HA-Pyr^1-466^. (B) *In vitro *transcription/translation of HA-Pyr^1-348 ^(left lane) and HA-Pyr^1-466 ^(right lane) blotted with anti-HA, show HA-Pyr^1-348 ^is likely post-translationally modified *in vivo*. (C) Schematic showing predicted events to explain the results in A and B: 1.) HA-Pyr^1-348 ^and HA-Pyr^1-466 ^are both translated to their predicted sizes as seen *in vitro *in (B). 2.) HA-Pyr^1-348 ^and HA-Pyr^1-466 ^are both modified post-translationally, likely by the addition of carbohydrate chains which increase their molecular weight to 50 kD and >50 kD (unobserved). 3.) HA-Pyr^1-466 ^is probably subsequently cleaved to decrease its molecular weight to 50 kD. Small branching trees represent modifications and the inverse triangle represents a predicted cleavage site.

In S2 cell supernatants, truncated Pyr constructs, HA-Pyr^1-348 ^and HA-Pyr^1-466 ^run at the same size on Western blots despite the larger construct containing 118 more amino acids (Fig. 6A). To understand if this was due to post-translational modifications we expressed both truncated constructs in a cell-free transcription/translation system and compared them to the size of the bands immunoprecipitated with anti-HA from the S2 cell supernatant. In S2 cells, HA-Ths^1-158 ^and HA-Pyr^1-466 ^ran similar to their predicted sizes (20 kD and 50 kD, respectively) but HA-Pyr^1-348 ^ran larger than its predicted size (at 50 kD instead of 35 kD), indicating that it may be glycosylated or otherwise modified (Fig. 6A). These modifications could correspond to the predicted O-glycosylation sites between aa 177 and 201 in Pyr (Fig. 1). When HA-Pyr^1-348 ^was expressed in the cell-free system (without the opportunity for post-translational modifications including glycosylation), it ran at its predicted size of 35 kD (Fig. 6B). HA-Pyr^1-466 ^likely contains the same modifications (and would likely also run larger than predicted, possibly around 65 kD), but we hypothesize it is subsequently cleaved to the smaller 49/50 kD size of secreted Pyr (Fig. 6C, predicted sequential steps 1-3). Carbohydrate modifications such as glycosylation can significantly affect the secretion, diffusion and binding capabilities of ligand proteins and the difference in modifications between Ths versus Pyr could contribute to their individual capabilities.

### Ths-Pyr chimeras reveal differences between Ths and Pyr C-termini

**Figure 7:**
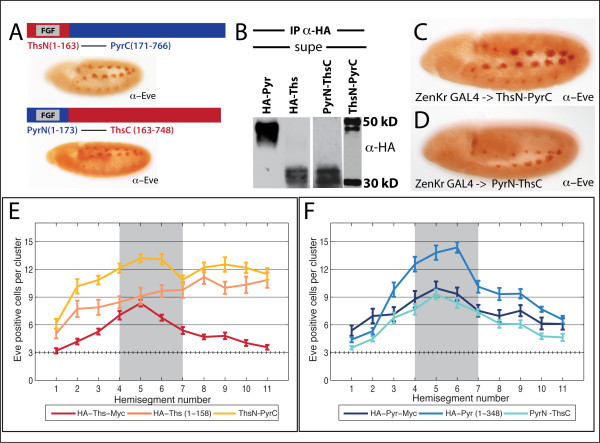
**Ths-Pyr Chimeras highlight inhibitory activity of Ths C-terminus**. (A) Schematics of ThsN-PyrC and PyrN-ThsC chimeric constructs and stage 11 embryos with 69B-GAL4 driving expression and function monitored with anti-Eve. Both chimeras give the +++Eve phenotype, meaning they support FGF activity. (B) Both chimeras are secreted, but cleaved differently in S2 cells. Supernatant immunoprecipitated with anti-HA and blotted with anti-HA, shows PyrN-ThsC is cleaved in a manner similar to Ths, while ThsN-PyrC may have both Pyr and Ths-derived cleavage sites. (C,D) Anti-Eve in stage 11 embryos with each chimera driven by ZenKr-GAL4 shows +++Eve cells in clusters outside the ZenKr domain for (C) pUASt-ThsN-PyrC and (D) pUASt-PyrN-ThsC. (E,F) Comparisons of Eve-positive cells per cluster in each hemisegment, scored and averaged as in Fig. 5. (E) ZenKr-GAL4 → ThsN-PyrC gives more Eve-positive cells than HA-Ths-Myc, suggesting that the Pyr C-terminus does not inhibit the Ths N-terminus the way that the Ths C-terminus does. (F) ZenKr-GAL4 → PyrN-ThsC has fewer Eve-cells than ZenKr-GAL4 → HA-Pyr^1-348^, and is similar to full-length HA-Pyr-Myc indicating the Ths C-terminus can likewise inhibit the Pyr N-terminus.

Results from the truncation constructs showed that eliminating the C-terminus from Ths has a different effect than eliminating the C-terminus from Pyr (see Fig. 5H,I). To further address the differences in the N- and C-termini of Ths and Pyr, we made chimeric proteins containing the N-terminus from one ligand and the C-terminus from the other, ThsN-PyrC and PyrN-ThsC. Both chimeras were secreted and functional, giving extra Eve-positive cells when driven by 69B-GAL4 (Fig. 7A). In S2 cell culture, both chimeras were processed and secreted as cleaved forms. When the N-terminus of each chimera is detected using an anti-HA antibody, we find that ThsN-PyrC is cleaved into a 50/52 kD doublet, and one band at 30 kD (Fig. 7B lane 4); as a result, it may contain cleavage sites and/or modifications derived from both Pyr and Ths. PyrN-ThsC is present as a small doublet around 30/35 kD, indicating it is likely cleaved and modified according to information derived from its Ths sequence (Fig. 7B lane 3 compared with lane 2). Importantly, this result shows that the cleavage is dependent upon the specific Ths or Pyr ligand sequence used and swapping sequence outside the FGF-domain allows us to see how the FGF-domain of one ligand acts in the context of processing like the other ligand.

The chimeras were also driven by ZenKr-GAL4 and the Eve-positive cells counted as was previously done for the truncated constructs. ThsN-PyrC supported more Eve-positive cells than HA-Ths and furthermore had the same altered profile as HA-Ths^1-158 ^(Fig. 7C,E). PyrN-ThsC had decreased Eve-positive cell clusters compared to the HA-Pyr^1-348^, and was similar to full-length Pyr (Fig. 7D,F). These results demonstrate that the Pyr C-terminus does not have the same inhibitory effect on the Ths N-terminus that the Ths C-terminus does.

### Deleting putative cleavage region renders Ths non-cleavable

**Figure 8:**
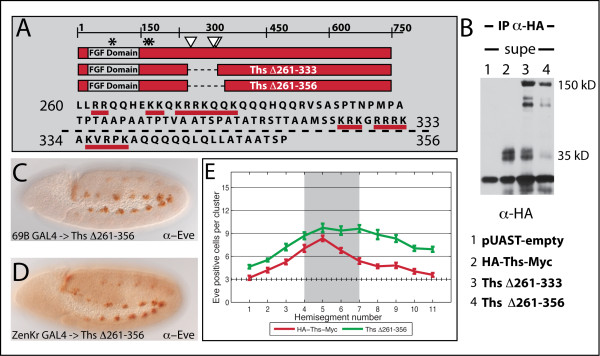
**Cleavage of Ths can be prevented through deletion of internal amino acids**. (A) Thisbe amino acid sequence deleted in Ths^Δ261-333^Myc, including 5 potential cleavage sites, underlined in red; below the dotted black line are the amino acids that differ between the two deletion constructs, Ths^Δ261-333^Myc and Ths^Δ261-356^Myc, including 1 additional potential cleavage site, underlined in red. (B) Western blot of anti-HA immunoprecipitations from supernatant of cells transfected with pUASt-empty, HA-Ths-Myc, HA-Ths^Δ261-333^Myc, and HA-Ths^Δ261-356^Myc. HA-Ths-Myc was loaded 5x less than the other samples to equalize the exposure while resolving the double-band at 35 kD. Ths^Δ261-333^Myc (lane 3) is still partially cleaved but has increased full-length protein and (lane 4) Ths^Δ261-356^Myc has less cleavage and more full-length product, as compared to HA-Ths-Myc (lane 2). (C) 69B-GAL4 driving Ths^Δ261-356^Myc results in more Eve-positive cells in every hemisegment, as compared to wild-type. (D) ZenKr GAL4 driving Ths^Δ261-356^Myc results in extra Eve-positive cells outside the source of expression. (E) Eve-positive cells counted in 11 hemisegments for 25 embryos and averaged as in Fig. 5. The gray box represents the source of expression supported by ZenKr-GAL4. As compared to HA-Ths-Myc, Ths^Δ261-356^Myc has a decreased gradient of functional output, resulting in a flatter profile.

In order to address whether the Ths cleavage is necessary for proper function, we attempted to engineer a Ths construct with a deletion of the putative cleavage sites to create an un-cleavable form of the ligand. To determine which region to delete, we considered the size of the 30-35 kD bands secreted in S2 cells and dibasic/multibasic Arginine/Lysine sequences characteristic of the recognition sites of furin-like proteases responsible for processing proproteins of other growth factors like Dpp into mature forms [43]. Sequences underlined with a red line (Fig. 8A) contain basic amino acids stretches of [R/K]-[X_n_]-[R/K] where × indicates any amino acid residue and *n *is 0, 2, 4, or 6, which is the consensus recognition sequence for furin-related proprotein convertases [43].

We deleted a 72 aa region containing 5 putative cleavage sites to generate the construct HA-Ths^Δ261-333 ^-Myc (Fig. 8A). When HA-Ths^Δ261-333^-Myc was immunoprecipitated from S2 cell culture with anti-HA antibody, the full-length band became much more prominent than that associated with HA-Ths-Myc (Fig. 8B, lane 3 vs. 2), although cleavage products still remain. We then extended the deleted region 23 aa further to remove one additional weak match to the furin consensus sequence to make HA-Ths^Δ261-356^-Myc. The cleaved bands were further diminished, although one band of ~35 kD was still detectable (Fig. 8A and 8B, lane 4). The simplest interpretation is that these 95 amino acids include most of the relevant cleavage sites for Ths, and deleting them results in a shift of the dominant Ths protein species to the full-length form.

The function of HA-Ths^Δ261-356^-Myc was tested using both the 69B-GAL4 assay and the ZenKr-GAL4 assay. We hypothesized that the drastic reduction of cleaved Ths and the presence of increased full-length Ths would result in either dramatically less function if cleavage were activating or dramatically more function if cleavage were inactivating. Surprisingly, the result was neither of these extremes, but instead HA-Ths^Δ261-356^-Myc was still able to generate supernumerary Eve positive cell clusters like the other constructs (Fig. 8C) and when the source of expression was limited using ZenKr-GAL4, the graded output of Eve positive cells was flattened as compared to HA-Ths-Myc (Fig. 8D, E), similar to the profile of HA-Ths^1-158^, yet not as potent an activator (compare with Fig. 5H).

Thus by deleting 95 aa, we can affect the proteolytic processing of the Ths protein such that the majority of the protein is present as full-length. Because this construct supports detectable activity, we suggest it is unlikely that cleavage is required for this activity. However, we cannot dismiss this possibility as some cleaved product is detected and the effective dose of FGFs is often very small; the remaining cleaved products in HA-Ths^Δ261-356^-Myc may be sufficient to function when overexpressed at high levels with GAL4 drivers. Nevertheless, HA-Ths^Δ261-356^-Myc supports an expanded profile similar to HA-Ths^1-158^; perhaps the flattened output is an indication that both of these proteins are not endocytosed properly (see Discussion). It is possible that cleavage is required before ligands can be effectively endocytosed from the extracellular space.

## Discussion

Proteolytic processing regulates many signaling molecules in both vertebrates and invertebrates, and unlocking the mechanisms behind regulatory cleavage events has been an on-going effort for each protein. In 1990, Dpp was first reported to be cleaved in S2 cells [45], and recent studies have continued to piece together the details of the maturation of the Dpp protein [28]. Our current analysis is the first evidence for cleavage of Ths and Pyr FGF ligands for the Htl FGFR. In this study we have shown that Ths and Pyr proteins are both cleaved and secreted from S2 cells and cleaved Ths and Pyr can be detected in embryonic extracts. Truncated ligands with the N-terminal FGF-domain are functional. Additionally, spatially restricting the source of FGF ligands and using domain-swapped chimeras revealed that the C-terminus of Ths has an inhibitory capability while the C-terminus of Pyr does not.

### The roles of Ths and Pyr C-terminal domains are different

The C-terminus of Ths, but not that of Pyr, can be detected extracellularly in tissue culture cells, suggesting that the processing situation for Ths is likely different to that for Pyr and raising the possibility that the function of the C-termini of these proteins has diverged. Even though we can detect cleaved forms of Ths inside the cell, indicating that cleavage likely occurs intracellularly, we can also detect both full-length and cleaved forms of Ths outside of the cell. Why would both forms be secreted if only the cleaved form was necessary to participate in receptor binding? In the case of Notch, two of the processed forms remain non-covalently associated and must be further processed for release [32]. In the case of Ths, the presence of both the unprocessed form and the processed form outside the cell creates the opportunity for an interaction.

While expression of full-length Ths has a graded range-of-action, we were surprised to find that truncated Ths has an extended range-of-action which suggests that the C-terminal domain is inhibitory. The mechanism by which the C-terminus of Ths accomplishes its inhibitory role remains an intriguing question. Does the C-terminus affect the rate of cleavage, the diffusion range, the rate of endocytosis, or might it physically interact with the N-terminus to directly inhibit FGF binding to the receptor? The Ths C-terminus has a similar effect on the Pyr FGF domain-containing N-terminus, as the PyrN-ThsC chimera is functionally restricted compared to the shorter Pyr^1-348^. In contrast, the Pyr C-terminus does not have the same effect on the Ths N-terminus, as ThsN-PyrC is highly potent and diffuses in an unrestricted manner, similar to truncated Ths^1-158^.

Our data supports the view that Pyr is processed inside the cell and that only the N-terminal cleaved form is released; therefore the Pyr C-terminus may only have a cell autonomous effect and likely does not affect the secreted protein directly once it is released. The local potency of HA-Pyr-Myc is less than the truncated Pyr constructs; so we propose cleavage of Pyr inside the cell may be a rate-limiting step.

Our results are also consistent with the idea that Pyramus could contain a transmembrane domain, as predicted, although our inability to follow the Pyr C-terminus has prevented us from confirming this prediction. Although we have not yet uncovered a specific role for the Pyr C-terminus, of note is the fact that the C-terminal domain of Pyr exhibits homology to the intracellular protein human protein Trinucleotide Repeat-Containing 15 (Tnrc15) implicated in Parkinson's disease (18% identity vs. 38% similarity over an approximately 500 aa stretch; data not shown) [49]. Tnrc15 interacts with Grb10, which, in turn, interacts with EGFR, MAP2K1 and many other signaling molecules [50]. This homology suggests the Pyr C-terminus may function inside the cell to regulate signaling, a function that likely is distinct from that of the Ths C-terminal domain.

### Processing of FGF ligands: proteolytic cleavage and post-translational modification

Further studies will be required to understand the role that processing of Ths and Pyr plays in the regulation of FGF signaling in *Drosophila*. The proteases responsible for the processing may themselves be spatially or temporally regulated at the transcriptional level, or separated into different subcellular compartments. Additional regulation of FGF signaling activity by proteases, which alter ligand activity and/or diffusion, could explain how FGFs are able to perform so many distinct functions in animals. For example, the diffusion range of Ths and Pyr, possibly regulated by proteolytic cleavage, could be important to support different functions. For instance, recently we have learned that during gastrulation Ths and Pyr guide the symmetric collapse of the mesoderm first and subsequently control intercalation of cells required for monolayer formation [51]. For collapse, a long-range signal might be required, whereas to support the small cell movements of intercalation a short-range signal may be more effective. In addition, findings from the TGF-β signaling family show in some cases ligands are differentially processed in a tissue-specific manner. Differential processing of BMP-4 by furin proprotein convertases results in multiple ligand forms that exhibit differences in stability and ability to act at a distance in *Xenopus *assays [52,53]. Recent results on the BMP-2/4 homolog, Dpp, have found not only is Dpp processed in a tissue-dependent manner but different cleavage products are also required to provide sufficient function for wing and leg versus gut development [28,54]. These examples highlight the importance of ligand processing as a key mechanism used by the cell to control ligand presentation and tissue-specific signaling.

### Range of action: diffusion and regulated endocytosis

Diffusion range is very important for most secreted signaling molecules, and this range may be influenced by post-translational modifications, proteolysis, or interactions with other secreted molecules. Recent work from zebrafish has shown that the common homolog of Ths and Pyr, FGF8, can act as a morphogen and spread from its source in the mid-hindbrain boundary by simple diffusion[55]. A slower-moving species of FGF8 was also detected, which is thought to be interacting with heparin sulfate proteoglycans (HSPGs) in the extracellular matrix. HSPGs are extracellular matrix and cell surface macromolecules that consist of a protein core to which heparin sulfate (HS) glycosaminoglycan (GAG) chains are attached. HSPGs are required as a co-receptor in vertebrate FGF signaling and might also be involved in *Drosophila *FGF signaling [56]. Alternatively, the excessive glycosylation implicated in the molecular weight of full-length Ths (150 kD compared to the predicted size of 82 kD) implies that the fully modified Ths molecule may likely be slow diffusing even without binding to HSPGs. Cleaved Ths might be freed from such glycosylation-mediated "inhibition" and allowed to diffuse farther and faster. The full-length and fully modified form may be protected from proteolysis by glycosylation [57], resulting in local FGF signaling, which may be preferred in some cases. Future studies will explore whether Ths and Pyr have different diffusion rates, and if these rates are affected by post-translational modification.

Furthermore, the gradient formed by the HA-Ths-Myc construct may be dependent on the uptake of ligand in a "source-sink" mechanism similar to what is observed for FGF8 diffusion in the zebrafish developing midbrain-hindbrain region [55]. In this scenario, cleavage could produce a form of Ths that is recognized and endocytosed, and may explain the more long-range activity associated with HA-Ths^Δ261-356^-Myc. Along these lines, short Ths^1-158 ^may be lacking such an internalization sequence to support the "flattened" output profile observed.

In the embryo, the switch between secretion of truncated or full-length ligand could be tissue-specific or temporally regulated as a means to support differential activity/range of the ligands. Once the proteases that process Ths and Pyr are uncovered, it will be possible to study the relationship between proteolysis and range-of-action.

### Implications for vertebrate studies

Lastly, this new molecular data on Ths and Pyr raise questions about the evolutionary history of the FGF family. All 24 FGF family members in vertebrates are relatively small proteins. Did Ths derive its long C-terminus in the *Drosophila *lineage indpendently before it was duplicated to produce Pyr, or was the ancestral FGF a long protein with cleavage sites that were lost in the vertebrate lineage? To obtain some insight into these questions, we can look to FGFs characterized in other animal models [58]. Worms have two FGF ligands, LET-756 and EGL-15. EGL-17 is small and LET-756 is 425 aa, an intermediate size, but not known to be cleaved [59,60]. Additionally, Bnl, the other FGF ligand in *Drosophila*, is approximately the same size as Ths and Pyr (i.e., 84 kD), although it is not more related to them than Ths/Pyr are to FGF8 [61]. Therefore, it seems most likely that the *Drosophila *genome tolerates the lengthening of proteins and has found secondary ways of processing them during post-translational regulation. This theory of differential genome tolerance was also put forth by Schmid and Tautz regarding *Drosophila *transcription factors, which are on average 30% longer than their *Tribolium *homologs [62]. Another possibility (which is not mutually exclusive) is that the *Drosophila *FGF ligands are multi-functional proteins, with the FGF-homologous portions responsible for activation of FGFRs and with the low-complexity regions (i.e., C-termini for Pyr and Ths) supporting additional functions, other than receptor-binding, required to support FGF signaling. Furthermore, while Ths and Pyr likely arose from an ancient duplication, [16], the C-termini of these proteins have diverged: the Pyr C-terminus is most similar to an intracellular protein (i.e., Tncr15 which interacts with the adaptor Grb10) and the Ths C-terminus exhibits homology to highly glycosylated proteins, likely found extracellularly. In vertebrates, studies on the Klotho protein suggest that at least some endocrine FGFs interact with additional proteins to influence receptor binding and activity [63]. Perhaps the *Drosophila *FGFs are ancestral multi-functional ligands that combine ligand-binding and Klotho-like adaptor or HSPG functions. In any case, whether these "long" FGFs are novel inventions of *Drosophilids *or ancient remnants of more ancestral FGFs, we contend that the modular nature of *Drosophila *FGFs may provide important insights into mechanisms that affect FGF activity, which is best examined by comparing the activities of the diverged ligands, Ths and Pyr.

## Conclusions

In the present study we have provided evidence for the proteolytic processing of Drosophila FGF ligands Ths and Pyr in both S2 cell culture and the embryo. Functional data was presented showing that truncated, FGF-domain-containing N-termini are capable of functioning in the embryo without their respective C-termini. Restricted ectopic expression *in vivo *demonstrated the difference in signaling capability between Ths and Pyr in embryos and domain-swapped chimeras highlighted the differences in the C-terminal domains of Ths and Pyr. These findings advance our understanding of the mechanism of FGF signaling in *Drosophila *and also suggest FGF signalling in *Drosophila *may be even more similar to that in vertebrates.

## Methods

### Prediction Programs

N-glycosylation sites in the *Drosophila *FGFs were predicted using the NetNGlyc Server version 1.0 at http://www.cbs.dtu.dk/services/NetNGlyc/[64]. O-glycosylation sites were predicted using OGPET version 1.0 prediction tool, ^© ^University of Texas at El Paso (UTEP) El Paso, TX accessed at http://ogpet.utep.edu/OGPET/contact.php.

### Fly Stocks and Constructs

69B-GAL4 (Brand and Perrimon, 1993) and zenVRE.Kr-GAL4 (Frasch, 1995) fly stocks have be previously described. All Ths and Pyr truncation, deletion and chimera constructs were inserted into the pUASt-attB vector. 1X HA tags were inserted by fusion PCR just after the N-terminal signal peptide, between amino acid 22 and 23 for Ths (i.e., ALCTV - HAtag - EDYVI) and between amino acid 30 and 31 for Pyr (i.e., ASAAK - HAtag - NVLTL). 6X Myc tags were inserted with Xho1 sites at the C-terminus just before the stop codon. HA-Ths^(1-158)^, HA-Ths^(1-261)^, HA-Ths^(1-403)^, HA-Pyr^(1-220)^, HA-Pyr^(1-348)^, HA-Pyr^(1-466) ^were all PCR amplified from full *length ths or pyr *template and cloned into pUASt-attB with Not1/Kpn1 sites (Ths constructs) or BglII/Xba (Pyr constructs) sites. Of note is the fact that a full-length *pyramus *cDNA has not been isolated to date, neither from cDNA libraries nor when primers are utilized to PCR amplify the predicted full-length gene from cDNA directly. Therefore, the *pyr *coding sequence in hand is a recombinant DNA molecule composed of 1 kB of cDNA sequence fused to ~1.3 kB of genomic sequence, based on the current genome prediction [18].

### S2 cell culture and transient transfection

Schneider cells (S2) obtained from the Drosophila Genomics Resource Center (DGRC) were maintained in a 25°C incubator in Schneider's Drosophila Medium (Invitrogen, #11720-067), supplemented with 10% Fetal Bovine Serum (USA Scientific, #9871-5200), Pencillin-streptamycin (dil 1:100), and Fungizone (Invitrogen, #15290018), and filter sterilized. Cells were passed with a 1:10 dilution every 4-5 days.

Effectene transfection reagent (Qiagen, #301425) was used to transiently transfect DNA into S2 cells. 10 ul Effectene reagent and 3.4 ul Enhancer were used with 1ug DNA in 100 ul EC Buffer. Cells were seeded into 6-well culture dishes at a concentration of 2 × 10^6 ^cells/well. 100 uM CuSO4 was added the day after transfection to induce the expression of the vectors from the metallothionine promoter. Supernatant and cell pellet fractions were harvested 2 days post transfection. Cells were lysed with a denaturing lysis buffer (1% SDS, 50 mM Tris, 5 mM EDTA, DTT, DNase and protease inhibitor cocktail). Complete Protease Inhibitor Cocktail (Roche) was added to the supernatant fractions.

### Immunoprecipitation and Western Blotting

Avidin-conjugated beads from Pierce were used to pull down HA and Myc tagged FGF constructs with a HA-biotin or Myc-biotin antibody. 250 ul of supernatant or cell pellet was combined with 50 ul of avidin agarose beads (Pierce #20219), 25 ul 10x RIPA buffer (500 mM Tris pH8, 1.5 M NaCl, 5% DOC, 1% SDS, 1-% NP-40, 10 mM DTT, 10X Roche Complete protease inhibitors, pH to 8.0) and 0.5 ug anti-HA-Biotin (rat, 3F10, Roche #12158167001) or .01 ug anti-c-Myc biotin conjugated antibody (mouse, 9E10, Sigma # B7554) and rocked overnight at 4°C. Beads were washed 3 times 5mins with 1X RIPA buffer, and proteins were released from the beads by boiling in 1x SDS sample buffer for 5mins.

Immunoprecipitation samples were run on 10% SDS-PAGE gels, transferred for 6mins with iblot (Invitrogen) and blocked for 1 hour at room temperature in 4% milk in TBS-T. Primary antibodies were used at the following dilutions: 1:1,000 anti-HA (mouse monoclonal, Covance, 16B12) or 1:10,000 anti-Myc (rabbit, AbCam 9106) 1 hour at room temperature. Blots were then washed 3 times 15mins in 1X TBS-T. Secondary antibodies conjugated to HRP were anti-mouse (Upstate, 1 hr. RT, 1:10,000) and anti-rabbit (Biorad, 20mins, 1:10,000). Blots were washed again 3 times 15mins in 1X TBS-T, and once for 5mins in H2O to remove Tween. Visualizer (Upstate) was used to develop the blots.

### Generating Site-Directed Transgenics and UAS-GAL4 mediated expression

Ths and Pyr constructs were cloned into the pUASt-attB vector[47]. Proper folding/secretion was assayed in S2 cell culture and by Western Blot before injection. Constructs were injected by site-directed transgenesis into the 86FB location on the third chromosome [47]; Rainbow Transgenics (Newbury Park, CA) performed most injections, some were done in-house.

### Immunostaining S2 cells

No. 1 1/2, 22 mm × 22 mm glass coverslips (Corning, #2870-22) were cleaned by soaking in HCl for 1hr. and rinsed thoroughly with dH_2_O. The coverslips were air dried and treated with 50 uL of 1 mg/mL concanavalin A (MP Biomedicals, #195283). Transiently transfected S2 cells were allowed to spread on the coverslips and attach to the ConA coating. The cells were fixed in 4% paraformaldehyde for 15mins, rinsed 3X with PBT (1X PBS + 1% Triton), and blocked in 5% Normal Goat Serum (Invitrogen) for 10mins. Primary antibodies were added in 5% block for 1 hr at RT, and then rinsed off. Antibodies used were: rabbit anti-GFP (1:1000, Invitrogen, #A111-22), mouse anti-HA (1:1000, Covance #16B12) and rabbit anti-Myc (1:10,000, AbCam #9106). Secondary antibodies (anti-rabbit 555, 1:500, Alexa Fluor, Invitrogen) were also added in 5% block for 1 hr at RT and rinsed off. Triton was rinsed off with two washes in H_2_O. Samples were mounted to slides with Vectashield Hardmount.

### Immunostaining Drosophila Embryos

3-6 hr embryos were collected and dechorinated in 50% Bleach for 3mins. The embryos were fixed in Heptane Fixing Solution [0.4 mL formaldehyde, 4 ml Heptane, 3.6 mL Fixing Buffer (10 mM KPO4, pH6.8, 15 mM NaCl, 45 mM KCl, 2 mM MgCl2)] for 12mins on an orbital shaker on high. The Heptane/Formaldehyde was removed and replaced with MeOH. The embryos were rinsed in MeOH 4 times and stored at -20°C for short term or -80°C for long term. Embryos were blocked in 1x western blocking reagent (Roche) for 30mins RT, and primary antibody incubations were performed overnight. Primary antibodies used were: anti-Even skipped rabbit (1:1000, M. Frasch) and anti-βgal rabbit (1:250, Molecular Probes). Secondary antibody was applied for 1-2 hrs. at RT: anti-rabbit 1:200 (Vectastain, Vector labs).

### *In vitro *transcription/translation

TnT T7/T3 Coupled Reticulocyte Lysate System (Promega, #L5010) was used with pBS-Pyr and pBS-Ths, incorporating S^35 ^Methionine, to assay the unmodified, full-length size of the proteins. Transcend™ Non-radioactive Translation Detection System (Promega, #L5070) containing biotinylated lysine in the Transcend tRNA was used in conjunction with the TnT Coupled Reticulocyte kit to transcribe and translate pUASt-HA-Pyr^(1-348) ^and pUASt-HA-Pyr^(1-466)^, which were subsequently run on 10% SDS-PAGE and detected with anti-HA by Western Blot.

## Authors' contributions

ST and AS conceived of the study and designed the experiments, which were performed by ST. ST and AS wrote the manuscript. All authors read and approved the final manuscript.
